# Impact of Neutrophil Extracellular Traps on Thrombosis Formation: New Findings and Future Perspective

**DOI:** 10.3389/fcimb.2022.910908

**Published:** 2022-05-31

**Authors:** Yilu Zhou, Zhendong Xu, Zhiqiang Liu

**Affiliations:** Department of Anesthesiology, Shanghai First Maternity and Infant Hospital, School of Medicine, Tongji University, Shanghai, China

**Keywords:** neutrophil, neutrophil extracellular traps, thrombosis, Innate immunity, thrombosis - immunology

## Abstract

Thrombotic diseases seriously endanger human health, neutrophils and neutrophil extracellular traps (NETs) play an important role in abnormal thrombus formation. NETs are extracellular structures released by neutrophils upon stimulation by pathogens. NETs include neutrophil elastase (NE), myeloperoxidase (MPO), cathepsin G and other active substances. The network structure provided by NETs can prevent the spread of pathogens and effectively kill and eliminate pathogens. However, the components of NETs can also abnormally activate the coagulation pathway and participate in the formation of pathological thrombi. This review aims to summarize the mechanisms of NETs formation in detail; the research progress of NETs in venous thrombosis, arterial thrombosis, acquired disease-associated thrombosis, sepsis coagulation disorder; as well as the strategies to target NETs in thrombosis prevention and treatment.

## Introduction

Neutrophil extracellular traps (NETs) are extracellular structures released by neutrophils under stimuli, such as pathogens, histones and bacteriostatic proteins (Laridan et al., 2019). NETs can confine pathogens *in situ*, prevent the systemic spread of pathogens and assist phagocytes in killing pathogens, which is considered to be another mechanism of action of neutrophils in innate immunity (Papayannopoulos, 2018). Because the network structure of NETs can provide a scaffold for the aggregation of red blood cells and platelets, and its components can also activate the coagulation pathway, NETs have recently been discovered to be involved in thrombosis formation (Noubouossie et al., 2019). This review elaborates the formation mechanism of NETs and their role in thrombosis formation and focuses on research progress in different kinds of thrombosis formation as well as related thrombosis prevention strategies.

## NET Formation

**Figure 1:**
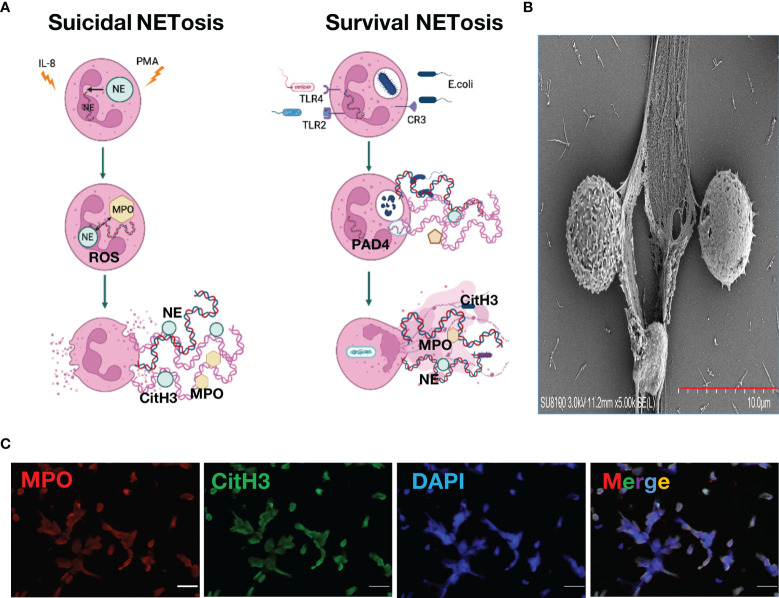
Neutrophil Extracellular Traps. **(A)**. Mechanism of neutrophil extracellular traps formation **(B)**. Neutrophil extracellular traps structure under scanning electron microscope **(C)**. Neutrophil extracellular traps stained with immunofluorescence, green, citrullinated histone H3; red, myeloperoxidase; blue, DAPI.

NETs were first named by Brinkmann et al. in 2004, but the mechanism by which NETs undergo NETosis has not yet been clarified (Brinkmann et al., 2004). According to differences in the pathogenic stimulation of neutrophils, activation of signaling pathways, and cell membrane integrity, NETs formation can be classified into two types: suicidal NETosis and survival NETs release (Thiam et al., 2020). In suicidal NETosis, neutrophils activate Fc receptors, Toll-like receptors (TLRs) and complement receptors under the stimulation of interleukin-8 and bacterial surface antigens. Activation of reduced coenzyme II oxidase leads to an increase in reactive oxygen species (ROS), and protein-arginine deiminase 4 (PAD4) further citrullinates chromatin after activation under the action of ROS, histone 3 and histone 4, causing DNA unwinding (Kapoor et al., 2018; Van Avondt and Hartl, 2018). ROS can also promote the release of myeloperoxidase (MPO) and neutrophil elastase (NE) in granules, and the NE can enter the nucleus to assist in the cleavage of histones and promote DNA unwinding (Jorch and Kubes, 2017). After the nucleus is ruptured, unwound DNA strands and histones enter the cytoplasm, and antibacterial proteins in the cytoplasm, such as MPO, CitH3, NE and cathepsin G, work together to form early NETs. At this time, neutrophils initiate the cell death program, and lysis of the cell membrane releases NETs (Kenny et al., 2017). In survival NETs release, neutrophils activate TLR-4 and TLR-2 receptors under stimuli such as bacterial lipopolysaccharide and gram-negative bacteria, resulting in PAD4 activation, and activated PAD4 enters the nucleus to citrullinate histone H3 and H4, causing DNA unwinding (Branzk et al., 2014; Thålin et al., 2019). Unlike suicidal NETosis, PAD4 is activated in a non-ROS-activated manner in surviving NETosis and is not accompanied by nuclear or cell membrane ruptures. Neutrophils themselves do not die, and unwound DNA strands bud into the cytoplasm and form early NETs with bacteriostatic proteins. They are exocytosed and released to the outside of the cell in the form of vesicles. At this time, although neutrophils have no nuclear DNA, they still have the ability to phagocytose and kill bacteria (Adrover et al., 2020). In the regulation of NETosis, pathogen fragment size and particle content in neutrophils can influence the generation and release of NETs (Branzk et al., 2014; Adrover et al., 2020). In addition, specific subpopulations of macrophages can also participate in the degradation of NETs through phagocytosis (Haider et al., 2020). Recent studies have also found that the pore-forming protein gasdermin D (GSDMD) can promote the formation and release of NETs by participating in the lysis of the neutrophil granule membrane, nuclear membrane and cell membrane and regulating the permeability of neutrophils. The intranuclear transfer of NE further affects NETosis (Sollberger et al., 2018). The link between NETosis and various kinases, such as cyclin-dependent kinases is also under investigation (Amulic et al., 2017; Wolach et al., 2018). However, there is still considerable controversy about whether PAD4 activation is necessary in NETosis and whether the DNA in NETs can be of mitochondrial origin (Lood et al., 2016; Claushuis et al., 2018). In conclusion, the mechanism of NETs generation and release still needs to be further explored (**Figures 1A–C**).

## NETs and Thrombosis Formation

**Figure 2:**
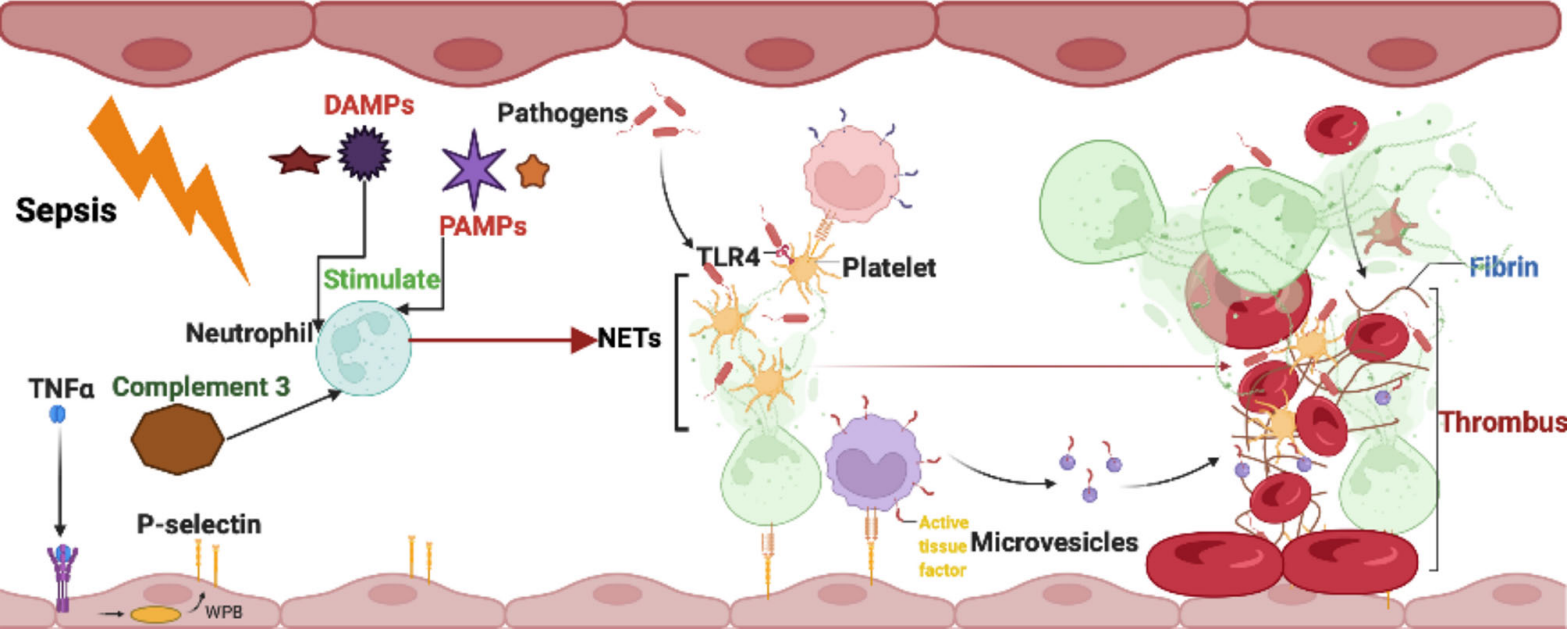
Interaction between NETs and other blood cells in thrombosis formation.

Thrombosis is an intravascular blood clot formed by substances such as platelets and coagulation factors in the case of vascular injury, abnormal blood flow or blood components and can play a role in repairing vascular injury and hemostasis under normal physiological conditions (Mackman et al., 2020). When the coagulation pathway is overactivated or fibrinolytic activity is decreased, the formation of thrombi will lead to vascular obstruction, resulting in a tissue blood supply disorder (Mackman et al., 2020). In addition, *in situ* thrombus shedding can also cause acute thromboembolism, such as acute myocardial infarction (AMI) (Wendelboe and Raskob, 2016), stroke (Farkas et al., 2019) with high fatality and disability rates. Several recent studies have found that neutrophils and NETs are ubiquitous in human arteriovenous thrombosis samples, mouse deep vein thrombosis (DVT) and other disease models (Wendelboe and Raskob, 2016; Noubouossie et al., 2017; Wang et al., 2018; Farkas et al., 2019). NETs have been further found to attract platelet activation and promote thrombosis by activating internal and external coagulation pathways, Generally, After vessel injury induced by neutrophils and NETs, TF under the endothelium is exposed to the vascular lumen and initiates extrinsic coagulation pathway. It serves as a cellular receptor for plasma factor VII/VIIa. Together, the factor VIIa : TF complex activates factor X and factor IX. Activated factor Xa in association with its cofactor Va, the prothrombinase complex, cleaves prothrombin to thrombin, which in turn cleaves fibrinogen to fibrin. Then, trans-glutaminase factor XIII cross-links fibrin to stabilize the thrombi (Noubouossie et al., 2017; Wang et al., 2018; Farkas et al., 2019). The network structure of NETs provides a scaffold for the deposition of platelets, erythrocytes, fibrinogen, von Willebrand factor (vWF) and other substances, such as platelet adhesion factors and extracellular bodies, which are conducive to thrombosis (Wang et al., 2018). Histones in NETs can attract platelet aggregation and activation through the interaction of fibrinogen, TLR2 and TLR4 and promote an increase in thrombin generation. In addition to the inhibition of activated protein C (APC), histones can also promote the expression of tissue factor on vascular endothelium and macrophages and promote coagulation (Yang et al., 2016; Noubouossie et al., 2017; Osada et al., 2017). NE and cathepsin G can promote the coagulation pathway induced by tissue factor and coagulation factor XII by hydrolyzing tissue factor pathway inhibitor (TFPI). Coagulation factor XII can also bind to surrounding platelets and DNA through its connection with NETs; it is directly activated by the coagulation pathway to expand coagulation (Vu et al., 2016; Döring et al., 2020). While cathepsin G promotes endothelial activation and prothrombin production, DNA inhibits fibrinolysis by forming complexes with fibrin and plasmin (Gould et al., 2015; Folco et al., 2018). In terms of the overall effect of NETs on thrombosis, NETs have been shown to promote the formation of erythrocyte-rich thrombi *in vitro*, and the erythrocytes in the thrombus are directly bound to NETs as observed by electron microscopy. In addition, NETs can interact with fibronectin and vWF to attract and promote platelet adhesion and activation (Gould et al., 2014; Martinod and Wagner, 2014). The connection between NETs and fibrinogen in thrombi can also promote fibrin deposition and improve thrombus stability; thus, thrombi containing NETs are more sensitive to tissue plasminogen activator (tPA) than thrombi with fibrin as the main component (Ducroux et al., 2018). The use of DNase I in mouse models can effectively prevent the formation of intravascular microthrombosis, which strongly suggests an effect of NETs on thrombosis (Jiménez-Alcázar et al., 2017). However, Noubouossie et al. (2017) pointed out that the promoting effect of NETs on thrombosis is achieved through its DNA and histone components rather than the NETs themselves. Therefore, evidence for a thrombopromoting effect of NETs as a whole remains to be further developed. Studies have shown that activated platelets can promote the formation and release of NETs through mechanisms such as P-selectin and high mobility group box protein 1 (Etulain et al., 2015; Kim and Lee, 2020). Therefore, the effects between NETosis and thrombosis may be reciprocal, and this mutually reinforcing cascade may play an important role in events such as thrombophilia in patients with thrombosis or an increased risk of thrombosis after infection (**Figure 2**).

## NETs and Venous Thrombosis

The relationship between NETs and venous thrombosis was first discovered in animal disease models (Wang et al., 2018). In the DVT disease model, staining of the thrombosed vein tissue indicated the presence of extracellular DNA; the DNA costained with vWF, and the serum DNA level was also increased, while the nonthrombotic vein tissue did not stain positive for extracellular DNA (Wang et al., 2018). A similar phenomenon was observed in a mouse DVT model, wherein activated neutrophils and NETs-specific markers, such as citrullinated histone H3 (CitH3) and extracellular DNA, were all present within the thrombus (Lim et al., 2018). Thus, the existence of NETs in venous thrombosis was preliminarily proven. In PAD4 knockout mice, NETosis is inhibited due to the inability of histones to be citrullinated under the action of PAD4 (Martinod et al., 2013). In addition, when using antibodies to inhibit neutrophil activation, DVT formation and thrombus diameter were significantly reduced in mice, while DNase-treated DVT mice had reduced thrombus recurrence and thrombus size (Mutua and Gershwin, 2021). Thus, the role of NETs in venous thrombosis has been clearly demonstrated. The relationship between NETs and venous thrombosis has also been demonstrated in human disease. First, the serum levels of DNA and activated neutrophil markers in DVT patients were higher than those in non-DVT patients, preliminarily verifying the association of NETs with venous thrombosis (Conteduca et al., 2022). The presence of extracellular DNA-histone complexes and neutrophils was further detected in thrombus samples obtained from DVT and PE patients by thrombectomy, and the different stages of thrombus formation were also detected by staining of thrombus cross-sections. The contents of neutrophils and CitH3 are different in the early stage of thrombosis; they are replaced by collagen fibers in the later stage, and the content of NETs markers is greatly reduced (Savchenko et al., 2014). *In vitro* experiments showed that NETs promote the differentiation of fibroblasts into collagen-secreting myofibroblasts, suggesting that NETs may be mainly involved in early venous thrombosis. With the development of thrombi, NETs can stimulate the production of collagen fibers from fibroblasts to promote maturation of the thrombus structure (Chrysanthopoulou et al., 2014). NETs have also been used as serum markers in the differential diagnosis of DVT, and different grades of extracellular DNA and CitH3 were detected in the serum of hundreds of DVT and non-DVT patients (van Montfoort et al., 2013). However, due to the limited sample size and differences in experimental techniques, the experimental results of NETs and venous thrombosis have been inconsistent across studies (van Montfoort et al., 2013; Savchenko et al., 2014; Chrysanthopoulou et al., 2014). Currently, there remains a lack of experimental evidence with a large sample size, which affects the role of NETs in venous thrombosis. The application of NETs, how to improve the stability of the results and further exploration of their role in thrombus structure need to be explored.

## NETs and Arterial Thrombosis

Unlike venous thrombosis, arterial thrombosis is more common in acute events resulting from thrombus shedding, such as AMI and ischemic stroke (Wendelboe and Raskob, 2016; Farkas et al., 2019). In this process, in addition to NETs *in situ* thrombi, the inflammatory response to tissue damage caused by ischemia can also recruit neutrophils (Farkas et al., 2019). Numerous neutrophils were found in tissue samples from acute myocardial infarction long before the discovery of NETs, and activated neutrophils were also found in thrombus samples obtained from patients with acute myocardial infarction (Bonaventura et al., 2020). Using NETs-specific markers and neutrophil antibodies, the widespread presence of NETs was further detected in thrombus samples and atherosclerotic plaques from patients with acute myocardial infarction, preliminarily demonstrating the existence of NETs in arterial thrombi (Riegger et al., 2016; Franck et al., 2018). Maugeri et al. (2014) also found the presence of NETs and platelet aggregation in coronary thrombi by immunostaining, verifying the attraction and aggregation of NETs to platelets. In addition, the content of NETs in recent coronary thrombi was higher than that in old coronary thrombi, and the content of NETs in coronary thrombi was positively correlated with the extent of myocardial infarction and the degree of ST-segment elevation (Mangold et al., 2015; Langseth et al., 2018). It was also positively correlated with the severity of prognostic outcomes in patients within two years, providing preliminary evidence for NETs as a disease prognostic indicator in myocardial infarction (Mangold et al., 2015; Langseth et al., 2018). In ischemic stroke, Laridan et al. (2017) detected the presence of NETs in thrombus samples. In a study of stroke thrombosis, NETs were found to be more abundant in cardiogenic and structurally mature old thrombi, and the content was positively correlated with the duration of thrombectomy and the number of surgical instruments used, which prompted the local microenvironment to affect NETosis (Boeckh-Behrens et al., 2016; De Meyer et al., 2017; Ducroux et al., 2018). In other arterial thrombi, such as intra-abdominal aortic aneurysm thrombi, DNA-histone complex-based assays have also suggested the presence of NETs (Fernández-Ruiz, 2018). It is worth noting that the content of NETs detected in thrombus samples from patients with acute myocardial infarction and ischemic stroke showed opposite results in thrombi of different maturities; by comparing arterial and venous thrombus samples, arterial thrombi with higher levels of DNA-histone complexes in NETs were found to have enhanced inflammatory responses and inflammatory cell recruitment within atheromatous plaques by promoting macrophage activation (Mangold et al., 2015; Warnatsch et al., 2015). NETs also affect the stability of atherosclerotic plaques by acting on smooth muscle cells through histones, resulting in reduced intraplaque smooth muscle cell content, reduced plaque interstitial collagen synthesis, and thinning of the fibrous cap, whereas PAD4 inhibition or anti-Ly6G can enhance the stability of plaques and the proportion of smooth muscle cells in plaques (Döring et al., 2020). Therefore, whether NETs may have different mechanisms of action in different etiologies and different origins of thrombi, whether the tissue microenvironment and vascular differences can modify NETs in thrombus formation, and whether NETs play a role in the early stage and development of arterial thrombosis need to be further explored.

## NETs and Anti-coagulant Lipid Syndrome (APS) and Tumor-Associated Thrombosis

Acquired thrombophilia refers to a group of clinical syndromes in which patients have a high risk of thromboembolism due to the presence of acquired thrombosis risk factors, and the embolism is mostly venous thromboembolism, including anti-coagulant lipid syndrome (APS) and tumor-associated thrombosis (Stevens et al., 2016). APS is an autoimmune disease characterized by recurrent venous or arterial thrombosis and miscarriage and is accompanied by increased serum antiphospholipid antibody (aPL) titers (Wirestam et al., 2019). Studies have shown that the content of extracellular DNA in the serum of patients with primary APS is increased, and the ability of isolated neutrophils to release NETs is enhanced, preliminarily verifying the existence of NETs in APS (Yalavarthi et al., 2015). In addition, the serum antibody of APS patients is positively correlated with the NETs content, and the isolated aPL mAb can enhance the release of NETs from neutrophils, suggesting that aPL-mediated NETs release may be a mechanism of APS-related thrombosis (van der Linden et al., 2018). In animal experiments, by adding aPL monoclonal antibody isolated from the serum of APS patients to DVT mice, the risk and degree of thrombosis were increased, the content of CitH3 in the thrombus was higher than that of the control group, and the level of serum DNA was also increased, validating the mechanism by which aPL promotes thrombosis by stimulating NETs release (Meng et al., 2017). The analysis of serum antibodies in APS patients further revealed the presence of anti-NETs antibodies, which led to a decrease in the degradation and clearance capacity of NETs formed in blood vessels, revealing another possible mechanism related to NETs in APS (Zuo et al., 2020).

The risk of thrombosis in cancer patients is higher than that in healthy people, which seriously affects the prognosis of patients (Fernandes et al., 2019). In addition to tumor-derived cytokines and chemotherapy-mediated tissue damage, the causes of thrombosis have also been found to be related to neutrophil activation and the formation and release of NETs (Hisada et al., 2015). In tumor patients, the contents of CitH3 and histone-DNA complexes in the systemic circulation and thrombus are increased, and they can be used to predict the risk of arterial thromboembolism in patients with malignant tumors of the lung and pancreas, suggesting that NETs play a role in tumor-related thrombosis (Oklu et al., 2017; Mauracher et al., 2018; Grilz et al., 2019). *In vitro* experiments have shown that human-derived pancreatic tumor cells can stimulate normal neutrophils to increase the release of NETs, and the NETs release capacity of neutrophils from gastric cancer patients is also enhanced compared to that of neutrophils from non-tumor patients (Yang et al., 2015; Abdol Razak et al., 2017). In animal experiments, the formation and release of NETs and the incidence of thrombi in tumor mice were higher than those in non-tumor mice, and CitH3 could be directly detected in the thrombus, further illustrating the relationship between NETs and tumor thrombosis (Hisada et al., 2020). Leal et al. (2017) observed increased formation and release of NETs when using tumor-derived granulocyte colony-stimulating factor (G-CSF) to stimulate neutrophils, first suggesting that NETs are involved in tumor thrombus formation. However, there is currently a lack of evidence on thrombosis after NETosis inhibition in acquired thrombophilia, and the evidence related to the impact of NETs on tumor prognosis needs to be further developed.

## NETs and Sepsis-Associated Coagulation Disorder

Sepsis is a systemic inflammatory response syndrome caused by infection (Singer et al., 2016). Patients with sepsis are often characterized by activation of the coagulation system, a progressive prothrombotic state, and dysregulation of the anticoagulation system, which may lead to disseminated intravascular coagulation (DIC), microvascular thrombosis, hypoperfusion and eventually multiple organ dysfunction syndrome and death (Semeraro et al., 2012; Tsao et al., 2015). A large amount of lipopolysaccharide is shed by bacteria in patients with sepsis; this is an important substance for activating neutrophils to produce NETs (Clark et al., 2007), which may be one of the reasons for the formation of NETs in sepsis. Platelets participate in the coagulation process and also play a role in inducing inflammation and resisting infection in infectious diseases (Thomas and Storey, 2015). Activated platelets during sepsis can directly sequester or kill pathogens or promote pathogen clearance by activating macrophages and neutrophils, and the specific receptor TLR4 is activated to promote NETs formation, ultimately causing platelet aggregation and microthrombosis (McDonald et al., 2012). Neutrophils isolated from the blood of patients with sepsis can release tissue factor (TF) through NETs, and this form of TF can induce thrombin generation *in vitro* and play a key role in the activation of the coagulation system in sepsis (Kambas et al., 2012). Plasma and platelets isolated from the blood of patients with sepsis can induce neutrophils to release NETs *in vitro*, and NETs produced by neutrophils from patients with sepsis have good procoagulant activity compared with healthy controls (Yang et al., 2017). NETs play an important role in thrombosis in septic patients through their interaction with platelets. Immune thrombosis refers to the innate immune response induced by thrombosis in blood vessels, especially in microvessels (Engelmann and Massberg, 2013). Immune thrombosis is supported by immune cells and specific thrombosis-related molecules and produces intravascular scaffolds that help identify and destroy pathogens, thereby protecting the body from pathogen-mediated damage (Loof et al., 2011). Neutrophils enhance thrombosis through a cell-specific mechanism of the immune response, and the NETs generated after activation have antibacterial and procoagulant activity (Kimball et al., 2016). NETs promote thrombosis in many ways and are a key factor in immune thrombosis (Goggs et al., 2020). Microvascular thrombotic diseases mainly include hemolytic uremic syndrome and thrombotic thrombocytopenic purpura (TTP), collectively referred to as thrombotic microangiopathy (TMA) (George and Nester, 2014). In hemolytic-uremic syndrome, in addition to promoting the inflammatory response and antibacterial effects, NETs also promote microvascular thrombosis, leading to renal failure in patients (Ramos et al., 2016; Leffler et al., 2017). In transplantation-related studies, elevated NETs levels significantly increased the risk of transplantation-related TMA (Arai et al., 2013), which may be due to the lack of DNase I, which can degrade NETs, in the plasma of these patients (Jiménez-Alcázar et al., 2015). ADAMTS13 is a plasma metalloproteinase that can cleave vWF and is a key enzyme in the diagnosis of TTP (Chauhan et al., 2006). In a recent study using exogenous recombinant human ADAMTS13 and DNase I-treated mice, the production of NETs *in vivo* was significantly reduced, indicating that ADAMTS13 may inhibit the formation of NETs and that DNase I can degrade NETs structures (Wong et al., 2020).

## NETs and Complement System in Thrombosis Formation

NETs formation is associated with systemic lupus erythematosus activity, and NETs can damage and kill endothelial cells and promote arterial inflammation of atherosclerotic plaques, thereby accelerating atherosclerosis in systemic lupus erythematosus (Barnado et al., 2016). Anti-phospholipid antibodies can stimulate neutrophils to produce NETs, which is closely related to thrombosis in patients with antiphospholipid antibody syndrome (Yalavarthi et al., 2015). The complement system is a part of the innate immune system composed of more than 30 proteins on the plasma and cell surfaces and is one of the main effector mechanisms of antibody-mediated immunity. Neutrophils are activated to release complement components, such as complement factor P, complement factor B, and C3, among which CFP is deposited on NETs and bacteria to induce formation of the membrane attack complex C5b (Yuen et al., 2016). In a mouse experiment with antiphospholipid antibody-induced thrombus formation, thrombus formation was significantly inhibited in complement component C3 knockout (C3-/-) and C5 knockout (C5-/-) mice, indicating that complement activation plays an important role in antiphospholipid antibody-induced thrombosis (Pierangeli et al., 2005). In another mouse experiment, complement C5 promoted liver injury associated with histone-induced lethal thrombosis (Mizuno et al., 2017). There is also an interaction between complement and platelets, which is manifested in the binding of factor Va to the C5b-induced binding site, promotion of its binding to factor Xa, and then binding to the functional prothrombin binding site on the platelet surface. These studies provide a basis for the study of the role of complement in thrombosis. In a spontaneous small intestinal tumor model, tumor development and hypercoagulability were both associated with neutrophils, especially the appearance of low-density neutrophils. Comparing the effect of complement C3a receptor (C3aR) to that in the absence of C3aR, NETs are more likely to be generated under the effect of C3aR, leading to the activation of coagulation in small intestinal tumors, a hypercoagulable state and thrombosis (Wiedmer et al., 1986). During a variety of bacterial infections, complement plays an important role while promoting the release of NETs; complement CR1 plays a leading role in this process, while complement CR3 plays an assisting role (Guglietta et al., 2016). NETs can serve as direct scaffolds for thrombosis and complement activation, and the opsonization and lytic activity of the complement system enhance the antibacterial properties of NETs. Although NETs, complement proteins, and coagulation factors work differently, they can still function in a large complex to protect the host from hemorrhage and infection; this cooperation is not limited to the site of injury but rather also occurs in the bloodstream. When this balance is disturbed, serious complications may occur, such as sepsis, deep vein thrombosis, autoimmune diseases and even cancer (Palmer et al., 2016). There is a close relationship between NETs and complement, and both play a role in the process of thrombosis. However, the specific division of labor of these components and whether there is a feedback mechanism still need further research.

## Potential Therapeutic Targets for NETs

The research on NETs in arteriovenous thrombosis and acquired thrombosis has provided a potential target for the treatment of thrombotic diseases. At present, research on NETs associated thrombosis has mainly focused on the promotion of NETs degradation and the inhibition of NETosis. Studies have shown that the use of DNase I can achieve partial lysis of NETs, and the combined use of tPA can achieve complete thrombolysis, which makes up for the defects of incomplete thrombolysis and low efficiency with tPA alone (Mangold et al., 2015). The use of DNase I in mice with thrombosis can also effectively prevent the recurrence of stroke, myocardial infarction and DVT (Ducroux et al., 2018). However, whether the degradation of NETs by DNase I will lead to the dispersion of components such as histones with procoagulant activity and increase the risk of thrombosis needs further study. Since APC can cleave NETs by cleaving histones, the strategy of using recombinant TMα to promote APC generation has also attracted researchers’ attention (Osada et al., 2017). Recent studies have also shown that the NE inhibitor can effectively reduce the generation of NETs in mice with endotoxic shock, providing another potential method for NETs-related antithrombotic therapy (Okeke et al., 2020). In terms of NETosis inhibition, thrombus formation was reduced in PAD4 knockout DVT mice, the PAD4 selective inhibitor GSK484 effectively inhibited NETosis *in vitro*, and there was no significant decrease in immune function in PAD4-deficient animal models. NETs are powerful targets in thrombosis therapy (Lewis et al., 2015; Martinod et al., 2015). Antioxidants, such as vitamin C, can affect NETosis by reducing the production of ROS, and vitamin C supplementation in septic mice can reduce the production of NETs; however, the specific effect of vitamin C on thrombosis has not been further studied in animal models and clinical trials. The use of JAK signaling pathway inhibitors to observe NETosis inhibition and thrombosis reduction in thrombosis mice needs to be further explored, and the efficacy of the related drug ruxolitinib in thrombosis prevention and treatment has also been confirmed in clinical experiments (Mohammed et al., 2013). The immunosuppressant colchicine has been proven in clinical and basic experiments to improve the prognosis of patients with myocardial infarction and stroke by inhibiting neutrophils, but its specific inhibitory effect on NETosis remains to be explored in depth (Vannucchi et al., 2015; Tardif et al., 2019). In addition, inhibiting the interaction of NETs and coagulation pathways can also achieve thrombosis prevention. Using the rhADAMTS13 protease to specifically cleave vWF, thrombolysis was observed in animal thrombosis models, and the combination of rhADAMTS13 and DNase I effectively improved myocardial infarction by reducing the interaction of vWF with neutrophils and NETs in myocardial tissue of mice (Kelly et al., 2018). The platelet receptor inhibitor P2Y12 and aspirin can also inhibit the interaction between platelets and NETs to reduce thrombosis formation (Li et al., 2017; Thiam et al., 2020).

## Conclusion and Future Perspectives

As a unique immune mechanism following the death of neutrophils, the role of NETs in thrombotic diseases has been verified in clinical samples and animal experiments, and research on NETs as antithrombotic therapy targets has been widely carried out. This study is expected to provide new insights into the treatment of existing thrombotic diseases and thrombotic complications. NETs-specific markers, such as CitH3 and the MPO-DNA complex, can also be used as biomarkers to provide the basis for the diagnosis or prognosis of thrombosis-related diseases and to explore the development and establishment of quantitative formulas for NETs-specific markers and NETs content in the circulation or local lesions. Methods such as improving the detection sensitivity of NETs can further provide new tools for precision medicine and outcome prediction. However, the results of existing studies have not been consistent, and there is still a lack of stronger experimental evidence for the actual effect of inhibiting NETs on promoting thrombolysis and preventing thrombosis. The issue of immunocompromised and increased bleeding risk also needs to be further explored. We look forward to future research on NETs to provide ideas and methods for solving thrombosis-related diseases.

## Author Contributions

All authors listed have made a substantial, direct, and intellectual contribution to the work and approved it for publication.

## Funding

This work was supported by the grants of Science and Technology Commission of Shanghai Municipality (20Y11901400) and Pudong Health Committee of Shanghai (PW2020D-13).

## Conflict of Interest

The authors declare that the research was conducted in the absence of any commercial or financial relationships that could be construed as a potential conflict of interest.

## Publisher’s Note

All claims expressed in this article are solely those of the authors and do not necessarily represent those of their affiliated organizations, or those of the publisher, the editors and the reviewers. Any product that may be evaluated in this article, or claim that may be made by its manufacturer, is not guaranteed or endorsed by the publisher.
